# “Lupus Myelitis” Revisited: A Retrospective Single-Center Study of Myelitis Associated With Rheumatologic Disease

**DOI:** 10.1212/NXI.0000000000200329

**Published:** 2024-10-23

**Authors:** Jonathan D. Krett, Angeliki G. Filippatou, Paula Barreras, Carlos A. Pardo, Allan C. Gelber, Elias S. Sotirchos

**Affiliations:** From the Division of Neuroimmunology and Neurological Infections (J.D.K., A.G.F., P.B., C.A.P., E.S.S.), Department of Neurology, Johns Hopkins University School of Medicine, Baltimore, MD; Multiple Sclerosis and Neuroimmunology Center (P.B.), Department of Neurology and Neurosurgery, Cedars-Sinai Medical Center, University of California, Los Angeles; and Division of Rheumatology (A.C.G.), Department of Medicine, Johns Hopkins University School of Medicine, Baltimore, MD.

## Abstract

**Background and Objectives:**

Previous reports of patients with myelitis associated with rheumatologic disease may have had unrecognized aquaporin-4 (AQP4)-IgG seropositive neuromyelitis optica spectrum disorder (NMOSD) or myelin oligodendrocyte glycoprotein (MOG)-IgG–associated disease (MOGAD). We clinicoradiologically and serologically characterized patients with myelitis associated with rheumatologic disease evaluated in the era of availability of MOG-IgG and more sensitive AQP4-IgG cell-based assays.

**Methods:**

A retrospective cohort (2018–2023) at Johns Hopkins Medicine with diagnoses of myelopathy and rheumatologic comorbidity was identified by electronic medical record (EMR) query. All patients with myelitis unrelated to typical multiple sclerosis (MS) were included and analyzed by chart review.

**Results:**

Of 238 patients identified by EMR query, 197 were excluded (148 not meeting prespecified inclusion criteria, 49 had typical MS), resulting in 41 patients for review. The mean age at myelitis onset was 44 ± 15 years; 39 (95%) were female. Rheumatologic diagnoses included 17 (41.5%) with systemic lupus erythematosus (SLE), 10 (24.3%) Sjögren syndrome (SS), 6 (15%) undifferentiated connective tissue disease (UCTD), 5 (12%) combinations of SLE/SS/UCTD with antiphospholipid antibody syndrome, 1 (2.4%) rheumatoid arthritis, 1 (2.4%) psoriatic arthritis, and 1 (2.4%) Behçet disease. 20 patients (49%) were diagnosed with AQP4-IgG seropositive NMOSD, 3 (7%) with MOGAD, and 18 (44%) had “double-seronegative” myelitis. Of these 18, 3 were diagnosed with AQP4-IgG seronegative NMOSD, 1 neuro-Behçet disease, and 14 other (unclassifiable) myelitis. Excluding 1 patient with neuro-Behçet disease, 18 (90%) of 20 AQP4-IgG seropositive patients had longitudinally extensive cord lesions compared with 5 (29%; *p* < 0.001) of 17 “double-seronegative” patients and 2 (67%) of 3 with MOGAD. “Double-seronegative” patients more commonly had CSF-restricted oligoclonal bands. Functional outcomes did not differ by diagnosis, and most patients received acute immunotherapy at the time of initial myelitis diagnosis with at least partial recovery over a median follow-up of 38 (interquartile range: 9–74) months.

**Discussion:**

Approximately half of our rheumatologic disease cohort with myelitis unrelated to MS had AQP4-IgG seropositive NMOSD while MOGAD accounted for a small but clinically relevant proportion of patients. Further research is needed to characterize myelitis etiology in patients who are seronegative for both AQP4-IgG and MOG-IgG.

## Introduction

In a seminal case series in 1968, Johnson and Richardson described neurologic manifestations in 24 people with systemic lupus erythematosus (SLE), which included 1 patient with paraplegia who eventually died of systemic complications.^1^ At autopsy, she was found to have longitudinally extensive myelopathy with degeneration of the white matter from the cervical to sacral segments, involving nearly the entire cord circumference.^1^ To this day, our knowledge of so-called “lupus myelitis” continues to be driven by case reports and case series, involving patients who present with myelitis (inflammatory myelopathy) in the context of clinical or serologic profiles satisfying diagnostic criteria for SLE or Sjögren syndrome (SS).^2–5^

Since the discovery of aquaporin-4 (AQP4)-IgG as a pathogenic biomarker in neuromyelitis optica spectrum disorder (NMOSD) 2 decades ago,^6–9^ it has been recognized that many patients with longitudinally extensive myelitis (LEM), optic neuritis (ON), and other core syndromes of NMOSD are seropositive for AQP4-IgG and bear striking resemblance neurologically to their counterparts with rheumatologic disease and LEM who were either not tested for AQP4-IgG or tested with older, less sensitive assays.^4,10–15^ AQP4-IgG, a highly specific NMOSD biomarker, can be detected in more than two-thirds of patients with rheumatologic disease who present with NMOSD-typical syndromes.^12,13,16^ Antinuclear antibody and SS-A antibody seropositivity is also more frequent in AQP4-IgG seropositive than AQP4-IgG seronegative NMOSD.^11^ Moreover, studies support that SLE genetic risk variants are associated with AQP4-IgG seropositive NMOSD.^17,18^

A further complicating matter is that patients with neurologic presentations associated with rheumatologic disease can have an admixture of underlying pathologies. These include immune-mediated, vascular-thrombotic (as in antiphospholipid antibody syndrome; APLS), toxic-metabolic related to medications or end-organ failure, and other pathologies.^19^ In recent years, myelin oligodendrocyte glycoprotein (MOG)-IgG–associated disease (MOGAD) has also emerged as a distinct autoimmune CNS demyelinating condition that can mimic NMOSD. Overlap with rheumatologic disease seems to be less prevalent with MOGAD compared with AQP4-IgG seropositive NMOSD,^10^ although a relatively high prevalence of MOG-IgG seropositivity (8%) has been reported in a single study of patients with SLE.^20^

Studies of myelitis associated with rheumatologic conditions largely predate the availability of MOG-IgG cell-based assays (CBAs)^21,22^ and widespread use of CBAs for detection of AQP4-IgG, which feature superior sensitivity and specificity compared with ELISA.^23,24^ Together with the establishment of NMOSD and MOGAD diagnostic criteria,^22,25^ along with canonical clinical and neuroimaging characteristics that can separate NMOSD and MOGAD from other mimics,^26–30^ clinicians practicing in the past 5–10 years have more precise tools for diagnosis than in previous decades. Timely and accurate diagnosis of AQP4-IgG seropositive NMOSD and MOGAD in people with rheumatologic disease is crucial because treatment strategies may diverge when comparing NMOSD, MOGAD, and (co-occurring) rheumatologic disease.^31–33^ Furthermore, given that previous studies have been confounded by lack of adequate evaluation for NMOSD and MOGAD, there is limited characterization of myelitis associated with rheumatologic disease occurring in the setting of dual MOG-IgG and AQP4-IgG seronegativity (“double-seronegative”).

In this context, we performed a retrospective study of patients with rheumatologic disease and myelitis who were evaluated at our center over the past 5 years, to determine clinicoradiologic and serologic overlap with AQP4-IgG seropositive NMOSD and MOGAD and further characterize cases of “double-seronegative” myelitis. We hypothesized that more sensitive AQP4-IgG assays and availability of MOG-IgG testing would identify AQP4-IgG seropositive NMOSD and MOGAD as prevalent causes of myelitis (particularly LEM) in patients with rheumatologic diseases.

## Methods

### Standard Protocol Approvals, Registrations, and Patient Consents

This study protocol was approved by the Institutional Review Board at the Johns Hopkins University School of Medicine (IRB00368901), with patient consent waived for this retrospective chart review.

### Identification of the Screening Population

To identify participants to be screened for eligibility, we queried electronic medical records (EMRs) of all patients evaluated at Johns Hopkins over a 5-year period from September 2018 through October 2023 with diagnoses of both myelopathy and a rheumatologic disease. The search was performed in October 2023. The full search strategy, including specific diagnostic terms used, is outlined in eMethods.

### Selection of Patients for Chart Review

**Figure 1:**
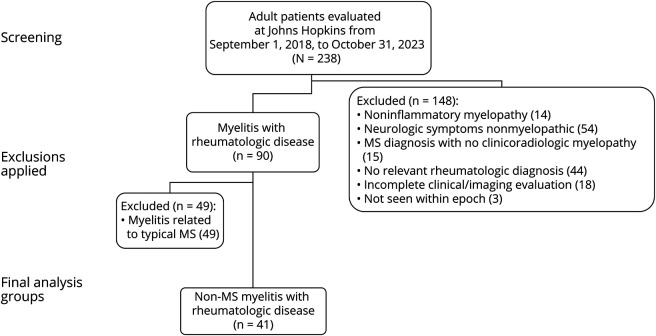
Flow Diagram Outlining Process for Including Patients With Rheumatologic Disease and Myelitis Evaluated at Johns Hopkins Hospital (2018–2023) MS = multiple sclerosis.

Inclusion and exclusion criteria were applied to patients identified by EMR query as presented in Figure 1. We included patients diagnosed with an inflammatory myelopathy (i.e., myelitis) and a documented rheumatologic condition. We also allowed patients to be included if there was concern for vascular myelopathy, as has been described in SLE/antiphospholipid syndrome/vasculitis.^34^ Patients with myelopathy related to clearly noninflammatory, nonvascular etiologies (e.g., compressive) or nonmyelopathic explanation for neurologic symptoms (e.g., arising from the brain, peripheral nervous system, or functional neurologic symptoms) were excluded. Patients with insufficient clinical or imaging (i.e., MRI) data to adequately characterize their phenotypes were excluded. Finally, because our research question focused primarily on characterizing myelitis cases with features of “lupus myelitis” and phenotypic overlap with NMOSD/MOGAD, we excluded patients who had myelitis that was clearly explained by a diagnosis of typical multiple sclerosis (MS). Such patients would be considered as having rheumatologic disease with comorbid MS, rather than “lupus myelitis,” supported by literature showing that MS and SLE are only weakly associated.^35^ We also assessed for postinfectious or parainfectious myelitis and myelitis temporally associated with preceding vaccination and determined whether patients could be classified under established diagnostic criteria for other neurologic diagnoses (e.g., neurosarcoidosis and neuro-Behçet disease) with a view to exclude these patients from subsequent comparisons. Clinical, laboratory, and imaging data for all patients were reviewed in detail by 2 neurologists with expertise in neuroinflammatory disorders and 1 rheumatologist.

### Data Collection and Variables

Demographic status, rheumatologic diagnosis, neurologic clinical features, baseline immunomodulatory/immunosuppressive treatments, acute therapy type, and timing from symptom onset were collected based on the first clinical presentation with available data (index presentation). If necessary, these data were collected from follow-up clinic notes describing the initial clinical presentation in retrospect. Outcomes were determined based on each of the index presentation and last available follow-up visit. MRI spine data were collected at the index presentation and next available follow-up (or last follow-up, based on scan availability) while MRI brain and CSF data were collected from the index presentation as available. All available AQP4-IgG and MOG-IgG assay results were recorded.

Particular variables of interest included the clinical pattern, severity, and recovery from episodes of myelitis, including level of disability at nadir pertaining to the index event and at follow-up. Overall disability was measured by retrospectively determining the modified Rankin Scale (mRS) while ambulatory and sphincteric disability were measured with the modified Aminoff and Logue Scale (mALS).^36^ We also analyzed the prevalence, type, and result of AQP4-IgG and MOG-IgG assays. MRI scans were reviewed to examine characteristics of brain and spinal cord lesions, including (1) longitudinal extent, (2) axial location, and (3) the presence of gadolinium enhancement. Longitudinally extensive lesions were defined as spanning ≥3 vertebral segments on sagittal MRI scans. Because the location of lesions on axial images often varied by spinal level and, in some cases, was obscured by motion artifact, each MRI scan was categorized based on the predominant pattern at most vertebral levels as follows: central cord (involving both gray matter and white matter), central gray matter only (H-sign), anterior gray matter (owl eyes), antero/posterolateral white matter, or dorsal columns. We applied established diagnostic criteria to classify patients as AQP4-IgG seropositive or seronegative for NMOSD^25^ or MOGAD.^22^ For the purpose of our analysis, patients seronegative for both AQP4-IgG and MOG-IgG without an established myelitis etiology were classified as being “double-seronegative.” Patients within the “double-seronegative” category who did not have a specific diagnosis were deemed to have “other myelitis.” We avoided the terms “idiopathic myelitis” and “idiopathic transverse myelitis,” which can be misleading and result in premature diagnostic closure.^37,38^ Rheumatologic diagnoses and serologies were extracted from the EMR. Where available, we determined whether the rheumatologic condition featured symptoms or laboratory findings suggesting activity (e.g., documentation of cutaneous manifestations, sicca symptoms, inflammatory arthritis, and hematologic manifestations) at the time of myelitis onset and generated a binary variable denoting active vs inactive rheumatologic disease. For patients with lupus specifically, we used established indices supported by the literature (SLEDA-I).^39^

### Statistical Analysis

Statistical analysis was performed using Stata version 18 (Statacorp LLC, College Station, TX). We compared patients by dividing them into 3 main diagnostic groups based primarily on serologic status: (1) AQP4-IgG seropositive NMOSD, (2) MOGAD, and (3) “double-seronegative.” Note that “double-seronegative” patients could include those diagnosed with AQP4-IgG seronegative NMOSD, which is a poorly understood entity. Clinical and demographic features of patients were reported as frequencies for categorical variables and using measures of central tendency (mean or median) and variance (SD or interquartile range) for continuous variables. Statistical comparisons of categorical variables were performed using the χ^2^ or Fisher exact test while continuous variables were compared using the Student pairwise *t* test as appropriate. 1 patient with a diagnosis of neuro-Behçet disease was excluded from the statistical comparisons so as to focus on comparing those with established diagnoses (AQP4-IgG seropositive NMOSD, MOGAD) with those without established diagnoses in the “double-seronegative” group.

### Data Availability

Anonymized data used for this study are available from the corresponding author on reasonable request, with the proper data-sharing agreements in place.

## Results

### Cohort Characteristics and Diagnoses

Of 238 patients identified by EMR query, 148 were excluded because they did not have an inflammatory (or vascular) myelopathy, confirmed rheumatologic diagnosis, or sufficient clinicoradiologic data available (Figure 1). 49 patients were excluded because of having features consistent with typical MS, resulting in 41 patients for review. Demographic characteristics are summarized in Table 1. Rheumatologic diagnoses included 17 (41.5%) with SLE, 10 (24.3%) SS, 6 (15%) undifferentiated connective tissue disease (UCTD), 5 (12%) combinations of SLE/SS/UCTD with APLS, 1 (2.4%) rheumatoid arthritis, 1 (2.4%) psoriatic arthritis, and 1 (2.4%) Behçet disease. Overall, 17 (44%) of 39 patients with sufficient data had evidence of active rheumatologic disease at the time of their myelitis presentation; 26 (63%) of 41 patients had known rheumatologic disease before presenting with myelitis. Additional breakdown of the subgroup of patients with SLE specifically can be found in eTable 1.

**Table 1:** Baseline Demographic Features and Diagnoses of Patients With Rheumatologic Disease and Myelitis Evaluated at Johns Hopkins Hospital (2018–2023), Subdivided by Main Diagnostic Groups

	AQP4-IgG+ NMOSD	MOGAD	Double-seronegative^a^	*p* Value
	N = 20	N = 3	N = 17	
Age at myelitis onset, mean (SD)	51 (15)	31 (15)	39 (12)	0.022
Sex				0.069
Female	20/20 (100%)	2/3 (67%)	16/17 (94%)	
Race				0.67
Black	10/20 (50%)	1/3 (33%)	6/17 (35%)	
Asian	1/20 (5%)	1/3 (33%)	3/17 (18%)	
White	8/20 (40%)	1/3 (33%)	6/17 (35%)	
Other	1/20 (5%)	0/3 (0%)	2/17 (12%)	
≥1 myelitis relapse				0.019
No	8/20 (40%)	2/3 (67%)	14/17 (82%)	
Yes	12/20 (60%)	1/3 (33%)	3/17 (18%)	
History of optic neuritis	9/20 (45%)	1/3 (33%)	1/17 (6%)	0.019
History of brainstem/area postrema syndrome	3/20 (15%)	0/3 (0%)	6/17 (35%)	0.28
Rheumatologic diagnosis				0.35
SLE	8/20 (40%)	1/3 (33%)	8/17 (47%)	
SS	6/20 (30%)	0/3 (0%)	4/17 (24%)	
UCTD	1/20 (5%)	1/3 (33%)	4/17 (24%)	
RA	1/20 (5%)	0/3 (0%)	0/17 (0%)	
PsA	0/20 (0%)	1/3 (33%)	0/17 (0%)	
SLE and APLS	2/20 (10%)	0/3 (0%)	1/17 (6%)	
SS And APLS	1/20 (5%)	0/3 (0%)	0/17 (0%)	
UCTD and APLS	1/20 (5%)	0/3 (0%)	0/17 (0%)	
Active rheumatologic symptoms/laboratory results at time of myelitis^b^	9/18 (50%)	1/3 (33%)	7/17 (41%)	0.80
Rheumatologic diagnosis predates myelitis	13/20 (65%)	2/3 (67%)	11/17 (65%)	1.00
Baseline maintenance immune therapy				0.32
None	12/19 (63%)	1/3 (33%)	11/17 (65%)	
Hydroxychloroquine	6/19 (32%)	1/3 (33%)	5/17 (29%)	
Azathioprine	0/19 (0%)	0/3 (0%)	1/17 (6%)	
Rituximab	1/19 (5%)	0/3 (0%)	0/17 (0%)	
Adalimumab	0/19 (0%)	1/3 (33%)	0/17 (0%)	
Maintenance immune therapy after index myelitis/at relapse				0.60
None	3/19 (16%)	0/3 (0%)	4/17 (24%)	
Hydroxychloroquine	2/19 (11%)	1/3 (33%)	2/17 (12%)	
Azathioprine	2/19 (11%)	0/3 (0%)	1/17 (6%)	
Mycophenolate mofetil	2/19 (11%)	0/3 (0%)	4/17 (24%)	
Corticosteroids	0/19 (0%)	0/3 (0%)	1/17 (6%)	
Rituximab	8/19 (42%)	1/3 (33%)	4/17 (24%)	
Combined oral immunosuppressant and rituximab	2/19 (11%)	0/3 (0%)	1/17 (6%)	
Secukinumab	0/19 (0%)	1/3 (33%)	0/17 (0%)	

Abbreviations: APLS = antiphospholipid antibody syndrome; AQP4 = aquaporin-4; MOGAD = myelin oligodendrocyte glycoprotein antibody–associated disease; NMOSD = neuromyelitis optica spectrum disorder; PsA = psoriatic arthritis; RA = rheumatoid arthritis; SLE = systemic lupus erythematosus; SS = Sjögren syndrome; UCTD = undifferentiated connective tissue disease.

Categorical variable frequencies are displayed as n/N (%) and continuous variables as mean (SD). *p* Value represents the result of the χ^2^/Fisher exact test or pairwise *t*-test as appropriate. Optic neuritis and brainstem syndromes preceded myelitis in all but 1 case in the double-seronegative group.

a Includes 3 patients with AQP4-IgG seronegative NMOSD and excludes 1 patient with neuro-Behçet disease with the remainder classified as “other myelitis” due to unclear diagnosis.

b Refers to documentation of active symptoms of SLE, SS, inflammatory arthritis and/or laboratory erythrocyte sedimentation rate elevation, C-reactive protein elevation, hypocomplementemia, or anemia/leukopenia/thrombocytopenia explained by connective tissue disease.

**Figure 2:**
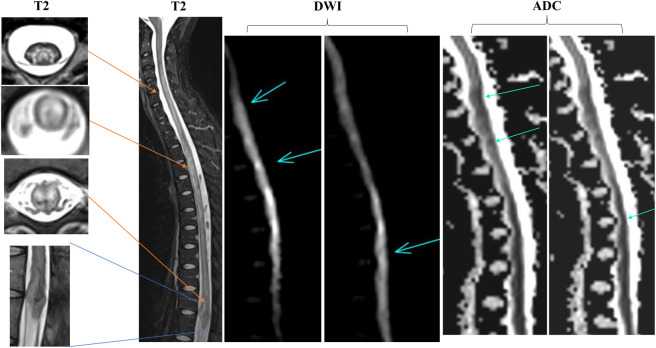
MRI Scans From a Patient With SLE and a Positive Anti-Cardiolipin Antibody With “Double-Seronegative” Myelitis The patient had an acute onset to nadir progressing to paraplegia within hours. DWI/DTI and ADC maps demonstrate focal areas of diffusion restriction (turquoise arrows) suggestive of infarct. T2 MRI sequences show an admixture of cord signal abnormalities that can be seen with both inflammatory myelopathy and spinal cord infarct, including an area of T2 hypointensity within the lumbar cord/conus suggestive of hemorrhage (blue lines; lower). No source for thromboembolism or primary vascular anomaly was found on the spinal MR angiogram. Serum AQP4-IgG and MOG-IgG were negative by live CBA. The CSF profile was inflammatory (WBC 65 cells/mm^3^, protein 66 mg/dL) with no oligoclonal bands, and there was poor response to acute immunomodulatory therapy with a mRS score of 4 at follow-up. ADC = apparent diffusion coefficient; AQP4 = aquaporin-4; CBA = cell-based assay; DTI = diffusion tensor imaging; DWI = diffusion-weighted imaging; MOG = myelin oligodendrocyte glycoprotein; mRS = modified Rankin Scale; SLE = systemic lupus erythematosus; WBC = white blood cell.

20 patients (49%) were diagnosed with AQP4-IgG seropositive NMOSD, 3 (7%) with AQP4-IgG seronegative NMOSD, 3 (7%) with MOGAD and 1 (2%) with neuro-Behçet disease, and 14 (34%) with other myelitis. All 3 patients with AQP4-IgG seronegative NMOSD met criteria because of having typical brainstem syndromes and compatible MRI brain findings, including area postrema syndrome and periependymal brainstem lesions concurrently with LEM. All 3 patients with AQP4-IgG seronegative NMOSD had a concurrent diagnosis of SLE while the 3 patients diagnosed with MOGAD had SLE, psoriatic arthritis, and UCTD, respectively. Two patients detailed in eFigure 1 and Figure 2 had mixed ischemic-inflammatory components to their myelopathy. No patients had a purely vascular noninflammatory myelopathy. Maintenance immune therapies at baseline, after index myelitis attack (nonrelapsing patients), or at the time of myelitis relapse are given in Table 1. Of note, 1 patient with psoriatic arthritis who developed myelitis due to MOGAD (MOG-IgG 1:1,000 by live CBA) was receiving the anti–tumor necrosis factor (TNF)-α agent adalimumab.

### Laboratory Testing

Most patients (n = 40/41, 98%) were tested at least once for serum AQP4-IgG, and 20 (49%) of 41 were positive by CBA or ELISA. The 1 patient without a documented AQP4-IgG test was presumed seronegative for the analysis. Of 21 AQP4-IgG seronegative patients, 6 were tested only by ELISA without any recorded CBA. Serum MOG-IgG testing was performed in 17 (41%) of 41 patients by CBA (56% fixed/44% live), with 4 (24%) of 17 positive, including one that was considered to be a false positive in the setting of borderline titer (1:20 by live CBA) and concurrent high-titer AQP4-IgG seropositivity (1:1,000 by live CBA), with clinical features consistent with AQP4-IgG seropositive NMOSD. Full neurologic and rheumatologic serology findings are detailed in eTable 2.

Seventeen (63%) of 27 patients in the total cohort with available CSF analysis had pleocytosis (7 polymorphonuclear, 8 lymphocytic predominant, 2 unknown). CSF-restricted oligoclonal bands (OCBs), where data were available, were present in 5 (42%) of 12 “double-seronegative” compared with 3 (25%) of 12 in the AQP4-IgG seropositive NMOSD group. When examining CSF white blood cell count collected within 30 days of myelitis onset, this was overall higher in “double-seronegative” patients compared with the AQP4-IgG seropositive NMOSD group (eTable 2; *p* = 0.34) but this was not statistically significant. The proportion with CSF glucose ≤50 mg/dL was greater in the “double-seronegative” group (6 of 10, 60%) vs AQP4-IgG seropositive NMOSD group (2 of 8, 25%; *p* = 0.36), but this difference was also not statistically significant. CSF MOG-IgG CBAs were performed in 4 patients with negative results in all cases (including 1 patient with MOGAD and serum MOG-IgG 1:1,000 by live CBA). Remaining CSF findings including OCB patterns can be found in eTable 2.

### Neurologic Clinical and Imaging Features at Myelitis Onset

**Figure 3:**
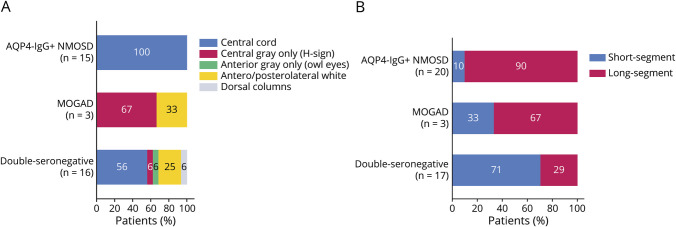
Bar Graphs Showing Distributions of MRI Spine Lesion Patterns by Main Diagnostic Groups Distributions of (A) axial patterns and (B) longitudinal extent on sagittal MRI are displayed as percentages along the x-axis with denominators shown next to diagnostic groups on the y-axis. Long-segment lesions were defined as those spanning ≥3 vertebral segments. AQP4 = aquaporin-4; MOGAD = myelin oligodendrocyte glycoprotein antibody–associated disease; NMOSD = neuromyelitis optica spectrum disorder.

Most (33 of 35 with available data; 94%) of the patients had an acute or subacute myelitis onset to nadir (6 hours–21 days) and were paraparetic or quadriparetic with at least antigravity strength (n = 29/39, 74%), without differences between AQP4-IgG seropositive NMOSD, MOGAD, or “double-seronegative” groups (Table 2, eTable 3). There were no differences in the frequency of flaccid vs spastic muscle tone at nadir by neurologic diagnostic group (eTable 3). As shown in Table 3, longitudinally extensive spinal cord lesions on MRI, as opposed to short-segment lesions, were more frequent in AQP4-IgG seropositive patients compared with the “double-seronegative” group (90% vs 29%, *p* < 0.001). Central cord lesions involving both gray matter and white matter were more common in AQP4-IgG seropositive vs “double-seronegative” patients (100% vs 56%, *p* < 0.001) while lesions in the antero/posterolateral spinal cord white matter (eccentrically located) were more common in the “double-seronegative” group (25% vs 0%, *p* < 0.001) (Table 3, Figure 3). Of the patients with MOGAD, 2 (67%) of 3 had longitudinally extensive lesions involving the central gray matter. Most patients (n = 29, 72%) had normal brain MRI or nonspecific T2 hyperintensities, without major differences by diagnostic groups. 3 of the patients in the “double-seronegative” group (3/17, 18%) had parenchymal T2 lesions on MRI brain, which were suspected to possibly be inflammatory based on location, size, and/or orientation but were not typical for MS, NMOSD, or MOGAD. Examples are shown in eFigure 2.

**Table 2:** Functional Outcomes at Myelitis Nadir, Acute Immunomodulatory Therapy, and Outcomes at Follow-Up in Patients With Rheumatologic Disease Evaluated at Johns Hopkins Hospital (2018–2023) by Main Diagnostic Groups

	AQP4-IgG+ NMOSD	MOGAD	Double-seronegative^a^	*p* Value
	N = 20	N = 3	N = 17	
Nadir antigravity strength present	15/20 (75%)	2/3 (67%)	12/16 (75%)	1.00
Nadir mRS score (IQR)	3 (2–4)	3 (2–4)	3 (2–4)	0.99
Nadir mALS gait score (IQR)	2 (2–5)	2 (0–4)	2 (2–5)	0.68
Nadir mALS sphincter score (IQR)	2 (0–3)	2 (0–2)	3 (2–3)	0.11
Acute treatment				0.24
None	1/20 (5%)	0/3 (0%)	3/17 (18%)	
Steroids	10/20 (50%)	2/3 (67%)	9/17 (53%)	
Steroids + PLEX	6/20 (30%)	1/3 (33%)	0/17 (0%)	
Steroids + PLEX + induction^b^	1/20 (5%)	0/3 (0%)	2/17 (12%)	
Steroids + induction^b^	2/20 (10%)	0/3 (0%)	3/17 (18%)	
Symptom onset to treatment, d (IQR)	2 (2–4)	5 (3–6)	5 (2–14)	0.27
Response after acute treatment				0.47
No response	0/19 (0%)	0/3 (0%)	2/15 (13%)	
Partial improvement	15/19 (79%)	2/3 (67%)	11/15 (73%)	
Complete resolution	4/19 (21%)	1/3 (33%)	2/15 (13%)	
Follow-up mRS score (IQR)	3 (1–4)	1 (1–1)	2 (1–3)	0.24
mRS improvement ≥1 nadir to follow-up	12/17 (71%)	2/2 (100%)	9/17 (53%)	0.45
Follow-up mALS gait score (IQR)	3 (0–5)	0 (0–0)	1 (0–3)	0.11
Follow-up mALS sphincter score (IQR)	2 (0–3)	0 (0–1)	1 (0–2)	0.49
Months of follow-up (IQR)	36 (10–80)	40 (6–74)	40 (9–67)	0.90

Abbreviations: AQP4 = aquaporin-4; IQR = interquartile range; mALS = modified Aminoff and Logue Scale; MOGAD = myelin oligodendrocyte glycoprotein antibody–associated disease; mRS = modified Rankin Scale; NMOSD = neuromyelitis optica spectrum disorder; PLEX = plasma exchange.

Categorical variable frequencies are displayed as n/N (%) and continuous variables as median (IQR). *p* Value represents results of the χ^2^/Fisher exact test as appropriate. “Sphincter” refers to bowel or bladder function.

a Includes 3 patients with AQP4-IgG seronegative NMOSD and excludes 1 patient with neuro-Behçet disease.

b Induction therapy included 7 patients receiving cyclophosphamide and 1 receiving rituximab.

**Table 3:** MRI Characteristics at Initial (Index) Presentation With Myelitis in Patients With Rheumatologic Disease Evaluated at Johns Hopkins Hospital (2018–2023), Comparing Results by Main Diagnostic Groups

MRI at index presentation	AQP4-IgG+ NMOSD	MOGAD	Double-seronegative^a^	*p* Value
	N = 20	N = 3	N = 17	
Cervical cord involvement	17/20 (85%)	2/3 (67%)	14/17 (82%)	0.68
Thoracic cord involvement	13/20 (65%)	3/3 (100%)	11/17 (65%)	0.76
Conus medullaris involvement	1/20 (5%)	0/3 (0%)	5/17 (29%)	0.12
Axial cord lesion pattern				<0.001
Central cord	15/15 (100%)	0/3 (0%)	9/16 (56%)	
Central gray only (H-sign)	0/15 (0%)	2/3 (67%)	1/16 (6%)	
Anterior gray only (owl eyes)	0/15 (0%)	0/3 (0%)	1/16 (6%)	
Antero/posterolateral white	0/15 (0%)	1/3 (33%)	4/16 (25%)	
Dorsal columns	0/15 (0%)	0/3 (0%)	1/16 (6%)	
Longitudinally extensive myelitis	18/20 (90%)	2/3 (67%)	5/17 (29%)	<0.001
Lesional enhancement	12/17 (71%)	1/3 (33%)	7/16 (44%)	0.22
Lesion focality				0.63
Monofocal	11/20 (55%)	2/3 (67%)	7/17 (41%)	
Multifocal	9/20 (45%)	1/3 (33%)	10/17 (59%)	
MRI brain findings				0.20
Normal/nonspecific	16/20 (80%)	2/3 (67%)	11/17 (65%)	
NMOSD-compatible	4/20 (20%)	0/3 (0%)	3/17 (18%)	
Other inflammatory	0/20 (0%)	1/3 (33%)	3/17 (18%)	

Abbreviations: AQP4 = aquaporin-4; MOGAD = myelin oligodendrocyte glycoprotein antibody–associated disease; NMOSD = neuromyelitis optica spectrum disorder.

Categorical variable frequencies are displayed as n/N (%). *p* Value represents the result of the χ^2^/Fisher exact test as appropriate. “Other inflammatory” MRI brain findings were T2-hyperintense lesions that were not typical for cerebral small vessel disease, multiple sclerosis, or NMOSD/MOGAD, but not entirely specific regarding etiology.

a Includes 3 patients with AQP4-IgG seronegative NMOSD and excludes 1 patient with neuro-Behçet disease.

**Figure 4:**
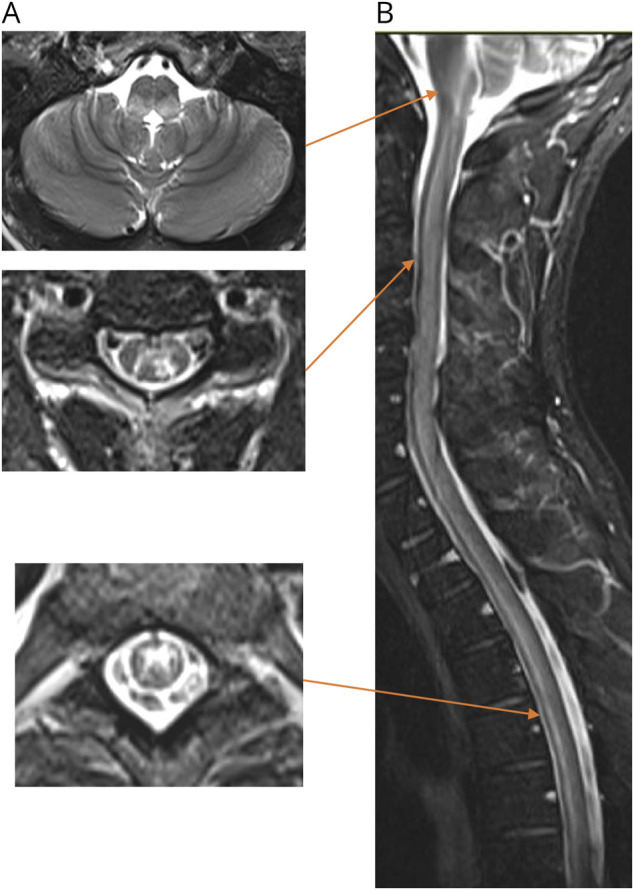
MRI Scans From a Patient With AQP4-IgG Seronegative NMOSD With Simultaneous Active SLE Symptoms included acute-onset paraparesis, urinary retention, nausea, oscillopsia, and intractable hiccups. T2-weighted (A) axial and (B) sagittal sequences show a dorsal periaqueductal medullary lesion contiguous with a longitudinally extensive spinal cord lesion. This lesion predominantly involved the central gray matter with a minority of posterolateral white matter (arrows). No lesional enhancement was seen (not shown). Testing for serum AQP4-IgG and MOG-IgG using both live and fixed CBAs was negative in both relapse and remission. AQP4 = aquaporin-4; CBA = cell-based assay; NMOSD = neuromyelitis optica spectrum disorder; SLE = systemic lupus erythematosus.

Figure 4 exemplifies imaging characteristics enabling diagnoses of AQP4-IgG seronegative NMOSD while Figure 2 demonstrates a patient with “double-seronegative” myelitis in the setting of SLE who had mixed inflammatory-ischemic components. eFigures 1 and 3 illustrate the heterogeneous presentations of “double-seronegative” myelitis in our cohort.

### Functional Outcomes, Treatment, and Imaging Follow-Up

At myelitis nadir during the index presentation, moderately severe disability was common in the overall sample (median mRS score of 3 [interquartile range; IQR 2–4]), without diagnostic group differences, and was also reflected in moderate gait and sphincteric dysfunction scores by mALS (Table 2, eFigure 4). Most of the patients received acute immune therapy, most typically high-dose IV corticosteroids with or without plasma exchange (PLEX). A greater proportion of patients in the AQP4-IgG seropositive NMOSD group received PLEX (6/20, 30%) compared with none in the “double-seronegative” group. After a median follow-up of 38 (IQR 9–74) months, the total cohort median mRS score was 2 (IQR 1–4), without statistically significant differences when comparing final mRS and mALS scores between subgroups (Table 2, eFigure 4), although there was a trend to better outcomes in the “double-seronegative” and MOGAD groups compared with AQP4-IgG seropositive NMOSD (eFigure 4). When examining change in the mRS score from the index presentation nadir to follow-up by subgroup, 12 (71%) of 17 patients with AQP4-IgG seropositive NMOSD, 9 (53%) of 17 “double-seronegative” patients, and 2 (100%) of 2 patients with MOGAD experienced at least a 1-point improvement in mRS.

Overall, 17 (41%) of 41 of the cohort had at least 1 relapse of myelitis during follow-up. The proportion with at least 1 relapse of myelitis was greater in the AQP4-IgG seropositive NMOSD group (12 of 20, 60%) compared with the “double-seronegative” group (3 of 17, 18%; *p* = 0.019) (Table 1). Myelitis relapses occurred in 1 of 3 patients with MOGAD (33%). Specific maintenance immune therapies at the time of myelitis relapse and those used during sustained relapse freedom thereafter are highlighted in eTable 4. Episodes of ON (unilateral or bilateral) all preceded initial myelitis presentations, with ON in 9 (45%) of 20 patients with AQP4-IgG seropositive NMOSD vs 1 (6%) of 17 “double-seronegative” patients (*p* = 0.011) and 1 (33%) of 3 patients with MOGAD (Table 1). Brainstem syndromes were less common and occurred in 3 (15%) of 20 AQP4-IgG seropositive patients and 3 (21%) of 14 “double-seronegative” patients who did not meet criteria for AQP4-IgG seronegative NMOSD.

MRI spine at follow-up was available for 30 patients at median 37 (IQR 6–91) months and did not show any findings discordant from patient diagnoses or index MRI (eTable 5). Of note, complete spinal cord lesion resolution comparing follow-up with index MRI occurred in 1 (50%) of 2 patients in the MOGAD group, 5 (36%) of 14 in the “double-seronegative” group, and 1 (7%) of 14 in the AQP4-IgG seropositive NMOSD group.

## Discussion

In this retrospective study of carefully selected patients with myelitis associated with rheumatologic disease (unrelated to typical MS), we found that nearly half had AQP4-IgG seropositive NMOSD while a small but clinically significant proportion had MOGAD. Most of the patients had either SLE or SS as their rheumatologic diagnosis. Dividing our cohort into diagnostic subgroups allowed for clinically relevant comparisons between “double-seronegative” myelitis and patients meeting diagnostic criteria for AQP4-IgG seropositive NMOSD and MOGAD. Other than 3 patients who met criteria for AQP4-IgG seronegative NMOSD and 1 with neuro-Behçet disease who was excluded from statistical comparisons, the “double-seronegative” group included mostly people with short-segment, eccentrically located spinal cord lesions on MRI, a higher proportion of CSF-restricted OCBs, and variable brain MRI findings. There were no significant differences in rheumatologic diagnoses represented or functional outcomes between subgroups. However, some intriguing trends emerged in the data including higher rates of PLEX for acute therapy in the AQP4-IgG seropositive group compared with “double-seronegative” patients, likely driven by suspected or established NMOSD diagnosis based on clinicoradiologic characteristics or history of ON in some patients. Patients with MOGAD in this rheumatologic disease cohort were also confirmed to have features which are often helpful to distinguish them from those with AQP4-IgG seropositive NMOSD including higher rates of exclusive gray matter involvement (H-sign) on spinal cord MRI and higher rates of lesion resolution on follow-up MRI.^28,40^

Our study builds on existing “lupus myelitis” literature, which mostly predates the contemporary AQP4-IgG and MOG-IgG testing context and based on case series/case reports.^3,4,14,41^ In one of the largest and relatively recent retrospective studies in 2009, the investigators reported findings from 22 patients with SLE and myelitis, dividing the cohort into 11 patients with “gray matter findings” and 11 with “white matter findings,” a distinction that was made based on clinical examination at presentation (i.e., flaccidity and hyporeflexia vs spasticity and hyperreflexia), rather than by imaging.^4^ They hypothesized that “gray matter” myelitis and “white matter” myelitis were distinct syndromes with different underlying pathomechanisms, with patients with “gray matter” myelitis having a more acute, treatment-refractory course with higher rates of CSF pleocytosis, lower CSF glucose, and lower rates of contrast enhancement on spine MRI.^4^ However, MOG-IgG testing was not available at the time of this 2009 study^4^ and AQP4-IgG testing was limited with 15 of 22 patients having documented AQP4-IgG assays. All AQP4-IgG testing in the preceding study was undertaken with tissue indirect immunofluorescence assays, which are less sensitive than both ELISA and CBA.^42^ Furthermore, AQP4-IgG seropositive patients were present in both groups (1 with “gray matter” myelitis and 4 with “white matter” myelitis), supporting that this purely syndromic definition of SLE-related myelitis is unlikely to reflect discrepant pathophysiologic underpinnings in the 2 clinically defined groups (i.e., “gray matter” and “white matter” myelitis).^4^ Owing to different diagnostic groups, CSF findings in our study are difficult to compare with those of the 2009 study summarized above. While a higher CSF WBC count and a greater proportion with low CSF glucose in the “double-seronegative” group were seen in our study, these findings were not statistically significant. Analyses were limited because of small sample size and since not all patients underwent CSF analysis in the acute phase. Based on the high proportion of patients in our study found to have a diagnosis of AQP4-IgG seropositive NMOSD or MOGAD, and that MOGAD can account for a substantial proportion of patients with pure spinal cord gray matter involvement on MRI,^30^ we view the “gray matter” vs “white matter” myelitis distinction as insufficient for use in contemporary clinical practice. There is an urgent need to update our definition of myelitis in the context of lupus and other rheumatologic diseases now that we have entered an era of defining neurologic diagnoses based on specific biomarkers (e.g., AQP4-IgG in NMOSD), which identify patients with unique prognoses, monitoring, and treatment requirements.^33^

In recent years, MOGAD has been established as a disease entity distinct from AQP4-IgG seropositive NMOSD, which is particularly important to identify given that patients with MOGAD generally recover more completely from attacks while approximately 50% of those with MOGAD may have nonrelapsing disease and consequently may not require long-term immunomodulatory therapy.^22,30,33^ Our findings are in keeping with literature suggesting that MOGAD is a less common overlapping neurologic diagnosis with rheumatologic disease than AQP4-IgG seropositive NMOSD,^10,43^ but it still occurred in 3 of 41 patients (among 17 tested for MOG-IgG).^44^ This reinforces the need to test patients presenting with an undifferentiated myelitis in the setting of rheumatologic disease for both AQP4-IgG and MOG-IgG to avoid misdiagnosis. We also identified 1 patient with a clinical phenotype typical of AQP4-IgG seropositive NMOSD with high-titer AQP4-IgG seropositivity who was concurrently borderline seropositive for MOG-IgG (a likely false positive), highlighting the value of scrutinizing clinical phenotypes together with assay specificity/sensitivity to make the correct diagnosis in patients with myelitis.^21,22,44^ Of interest, 1 patient with MOGAD had anti–TNF-α exposure at the time of their myelitis. Exposure to anti–TNF-α agents has been associated with an increased risk of MS onset and has been associated with MOGAD rarely.^45,46^

Our study helps to advance knowledge regarding potential etiologies underlying inflammatory myelopathies in patients who are seronegative for both AQP4-IgG and MOG-IgG (“double-seronegative”), without characteristics typical for MS or other well-established neuroimmune disorders. Evaluating and managing these patients in practice is challenging because the prognosis for risk of relapse along with choice of acute and maintenance immunomodulatory therapy is often unclear. For example, AQP4-IgG seropositive NMOSD can result in permanent neurologic disability when not treated early (within days of onset), with early PLEX proposed to be beneficial together with high-dose corticosteroids.^47,48^ If a patient with SLE presents with disabling myelitis and is not recognized as having AQP4-IgG seropositive NMOSD, the same patient may be treated instead with an induction course of cyclophosphamide after failing first-line therapy, which has mixed results regarding effectiveness in NMOSD.^49–51^ The first choice of maintenance immunomodulatory therapy for many patients with AQP4-IgG seropositive NMOSD is often rituximab (a B cell–depleting agent), or one of the newer FDA-approved therapies,^31^ whereas a common choice for those with SLE and neurologic disease is often a less selective immunosuppressant (e.g., mycophenolate mofetil). Selecting the appropriate maintenance therapy for the underlying neurologic disease is key because essentially all NMOSD-related and MOGAD-related disabilities seem to be mediated by relapses.^52^ Germane to this point is the current controversy regarding classification of “double-seronegative” myelitis with characteristics falling within the NMO spectrum because it is possible that this group may include patients with false-negative AQP4-IgG or MOG-IgG testing (due to issues such as timing, effects of treatment, and/or assay sensitivity), patients with alternative diagnoses (such as atypical MS or neurosarcoidosis), those with mimicking conditions (e.g., biotinidase deficiency), and/or those with distinct diseases associated with yet-to-be classified auto-antibodies.^25,53,54^ Our study suggests that in the rheumatologic disease population, there is a need to avoid anchoring on inflammatory causes because we screened out several patients with structural or neoplastic myelopathies and found 2 patients in our cohort who had mixed inflammatory-ischemic myelopathy in the context of active rheumatologic disease. By extension, it is likely that in the absence of AQP4-IgG and MOG-IgG seropositivity, some patients with “seronegative” NMOSD may have noninflammatory etiologies, and thorough exclusion of these causes is required.

A limitation of our study is that several patients had suboptimal evaluation with AQP4-IgG and MOG-IgG assays, which might have led to some patients in the “double-seronegative” group being misclassified. Considering that 6 patients in the AQP4-IgG seronegative subgroup were tested with less sensitive ELISA, it is possible that the proportion of patients with AQP4-IgG seropositive NMOSD is higher than we observed, particularly among those with LEM and other NMOSD-typical features. In addition, only 17 of 41 patients were tested for MOG-IgG, which may have been because some patients underwent their initial evaluation for myelopathy before being assessed at our center between 2018 and 2023 and before MOG-IgG assays became more widely available in 2017. Similarly, MOG-IgG testing was often performed remote from the index myelitis episodes (eTable 2), and seroreversion to MOG-IgG seronegative status over time is known to occur in MOGAD and may confound interpretation of test results.^55,56^ Furthermore, isolated CSF MOG-IgG seropositivity has been reported to occur in a subset of patients with MOGAD.^57–59^ In this cohort, CSF MOG-IgG testing was limited to only 4 patients and was negative in all cases (including 1 patient with a diagnosis of MOGAD based on serum testing). These issues highlight that the prevalence of MOGAD in our cohort may be underestimated and may actually account for a larger proportion of myelitis in people with rheumatologic disease. Finally, although we excluded patients with typical MS, it is conceivable that the higher rate of short-segment, eccentrically located spinal cord lesions and CSF-restricted oligoclonal bands in the “double-seronegative” group is due to some patients who had atypical MS with a paucity of brain lesions (i.e., spinal cord–predominant MS).

Overall, our study sheds light on underlying etiologies of myelitis in patients with rheumatologic disease and provides much-needed phenotyping of “double-seronegative” myelitis using imaging and contemporary serum biomarkers. Retrospective studies such as ours provide useful background information for designing prospective studies that can apply stricter selection of cases using modern biomarkers (particularly AQP4-IgG and MOG-IgG) and may be instructive for future multicenter registries, clinical trials, and new iterations of diagnostic criteria, which are the bedrock of our clinical practice in this patient population. Such studies will pave the way for more precise pathophysiologic diagnosis and, hopefully, the development of safer and more selective treatments for patients with myelitis and associated rheumatologic disease.

## Appendix Authors

**Table:**

Name	Location	Contribution
Jonathan D. Krett, MD	Division of Neuroimmunology and Neurological Infections, Department of Neurology, Johns Hopkins University School of Medicine	Drafting/revision of the manuscript for content, including medical writing for content; major role in the acquisition of data; study concept or design; analysis or interpretation of data
Angeliki G. Filippatou, MD	Division of Neuroimmunology and Neurological Infections, Department of Neurology, Johns Hopkins University School of Medicine	Drafting/revision of the manuscript for content, including medical writing for content; major role in the acquisition of data; analysis or interpretation of data; additional contributions (in addition to one or more of the above criteria)
Paula Barreras, MD	Division of Neuroimmunology and Neurological Infections, Department of Neurology, Johns Hopkins University School of Medicine, Department of Neurology and Neurosurgery, Multiple Sclerosis and Neuroimmunology Center, Cedars-Sinai Medical Center, University of California, Los Angeles	Drafting/revision of the manuscript for content, including medical writing for content; study concept or design
Carlos A. Pardo, MD	Division of Neuroimmunology and Neurological Infections, Department of Neurology, Johns Hopkins University School of Medicine	Analysis or interpretation of data
Allan C. Gelber, MD, PhD, MPH	Division of Rheumatology, Department of Medicine, Johns Hopkins University School of Medicine	Drafting/revision of the manuscript for content, including medical writing for content; study concept or design; analysis or interpretation of data
Elias S. Sotirchos, MD	Division of Neuroimmunology and Neurological Infections, Department of Neurology, Johns Hopkins University School of Medicine	Drafting/revision of the manuscript for content, including medical writing for content; major role in the acquisition of data; study concept or design; analysis or interpretation of data

## Glossary

CBA: cell-based assay
EMR: electronic medical record
IQR: interquartile range
LEM: longitudinally extensive myelitis
MOG: myelin oligodendrocyte glycoprotein
MOGAD: myelin oligodendrocyte glycoprotein–associated disease
mRS: modified Rankin Scale
MS: multiple sclerosis
NMOSD: neuromyelitis optica spectrum disorder
OCB: oligoclonal band
ON: optic neuritis
PLEX: plasma exchange
SLE: systemic lupus erythematosus
SS: Sjögren syndrome
UCTD: undifferentiated connective tissue disease
WBC: white blood cell
